# Current State of EEG/ERP Microstate Research

**DOI:** 10.1007/s10548-024-01037-3

**Published:** 2024-02-13

**Authors:** Christoph M. Michel, Lucie Brechet, Bastian Schiller, Thomas Koenig

**Affiliations:** 1 Functional Brain Mapping Lab, Department of Basic Neurosciences, Medical Faculty, University of Geneva, Geneva, Switzerland; 2 Center for Biomedical Imaging (CIBM), Lausanne, Geneva, Switzerland; 3 Department of Readaptation and Geriatrics, Medical Faculty, University of Geneva, Geneva, Switzerland; 4 Laboratory for Biological Psychology, Clinical Psychology, and Psychotherapy, Albert-Ludwigs-University of Freiburg, Freiburg, Germany; 5 Translational Research Center, University Hospital of Psychiatry, University of Bern, Bern, Switzerland

**Keywords:** EEG, ERP, Microstates, Resting states, Consciousness, Psychiatric diseases, Complexity, Brain functions

## Abstract

The analysis of EEG microstates for investigating rapid whole-brain network dynamics during rest and tasks has become a standard practice in the EEG research community, leading to a substantial increase in publications across various affective, cognitive, social and clinical neuroscience domains. Recognizing the growing significance of this analytical method, the authors aim to provide the microstate research community with a comprehensive discussion on methodological standards, unresolved questions, and the functional relevance of EEG microstates. In August 2022, a conference was hosted in Bern, Switzerland, which brought together many researchers from 19 countries. During the conference, researchers gave scientific presentations and engaged in roundtable discussions aiming at establishing steps toward standardizing EEG microstate analysis methods. Encouraged by the conference’s success, a special issue was launched in *Brain Topography* to compile the current state-of-the-art in EEG microstate research, encompassing methodological advancements, experimental findings, and clinical applications. The call for submissions for the special issue garnered 48 contributions from researchers worldwide, spanning reviews, meta-analyses, tutorials, and experimental studies. Following a rigorous peer-review process, 33 papers were accepted whose findings we will comprehensively discuss in this Editorial.

## Introduction

In 1987, Dietrich Lehmann, in collaboration with Hisaki Ozaki and Ivan Pal, published a seminal paper titled “*EEG Alpha map series: brain micro-states by space-oriented adaptive segmentation*” (Lehmann et al. 1987). This paper is widely acknowledged as the “birth certificate of EEG microstates”. Notably, as early as 1971, Lehmann had observed that “the maxima of the EEG alpha fields remain at one electrode position for a relatively long time compared to the time spent in transit from one preferred position to the next” (p. 447) (Lehmann 1971). He also used the term “microstate” in a book chapter written in German in 1984, concluding that “as long as the field configuration is the same, the same functional resting state can persist” (Lehmann 1984)(p.54). The discovery of temporally stable potential fields in spontaneous EEG and their potential connection to functional resting states of the brain traces its roots back over 53 years. Remarkably, this spans half the time since the initial unveiling of the EEG by Hans Berger (Berger 1929). However, during this early period, spatial analysis of EEG (commonly referred to as EEG mapping) was not widely embraced in the EEG community, which favored time-series-oriented pattern recognition and frequency analysis of individual channels, proving successful in clinical neurophysiology among other domains. It was not until the late 1990s that EEG microstate analysis, incorporating the spatial properties of potential fields, gained wider recognition. Interestingly, during this period, Bharat Biswal reported correlations between low-frequency fluctuations in blood oxygenation level depend (BOLD) signals in various motor brain regions, measured using fMRI, interpreting them as a manifestation of functional connectivity at rest. This phenomenon later became known as the resting-state networks (RSN) (Biswal et al. 1995).

Despite the inherently slow nature of BOLD signals, fMRI-based resting-state analysis has emerged as a crucial tool in the affective, cognitive, social and clinical neurosciences (Uddin et al. 2019). However, the recognition that functional brain networks need to exhibit fluctuations on a sub-second time scale to support complex mental activities and respond effectively to rapidly changing input (Bressler 1995) has underscored the necessity for methods capable of capturing such rapid dynamics. The EEG microstate approach proves to be well-suited for this purpose, as EEG microstates typically endure for 30–120 milliseconds. The advent of new methods for extracting EEG microstates (Pascual-Marqui et al. 1995), findings demonstrating the stability of EEG microstate topographies across different age groups in extensive datasets (Koenig et al. 2002), and initial reports highlighting microstate deviations in various diseases (Strik et al. 1995, 1997; Dierks et al. 1997; Koenig et al. 1999), collectively contributed to the increased adoption of EEG microstate analytics. This trend saw a significant surge, particularly following the publication of our review article in 2018 (Michel and Koenig 2018). While only five EEG microstate papers were published in 2010, the landscape has rapidly evolved, with a remarkable increase to 70 publications in 2022 (see figures in this special issue by (Kleinert et al. 2023a; Schiller et al. 2023; Tarailis et al. 2023; Zanesco 2023).

The increasing popularity of the EEG microstate approach motivated us to organize a dedicated conference focused on EEG microstates. The “50 Years of Microstates: Present State and Future Directions” conference took place in Bern, Switzerland, in August 2022, attracting many participants who presented their work and engaged in roundtable discussions. Encouraged by the success of this event, we initiated a call for a special issue on EEG and ERP microstate research to be featured in *Brain Topography*. The response was remarkable, with a total of 48 papers submitted for consideration. Following a comprehensive peer-review process, 33 out of the 48 submitted papers have been accepted for publication in this 3-part special issue (Table 1). Among them are 2 review papers, 3 meta-analyses, 2 tutorials, and 26 research articles. These research articles address methodological aspects (6 papers), microstates in various states of consciousness (5 papers), changes in microstates associated with psychiatric and neurological diseases (10 papers), and the influence of cognitive tasks on EEG/ERP microstates (5 papers). In the subsequent sections, we concisely summarize the primary findings reported in these publications.

**Table 1:** Publications in the Special Issue on EEG/ERP Microstates

First Author	Title	Country of author affiliations	Number of participants	Number of electrodes	Number of microstates	Analysis Software
Tarailis P	The functional aspects of resting EEG microstates: a systematic review	Lithuania, Switzerland	Review of 50 studies	Variable	7	Variable
Schiller B	EEG microstates in social and affective neuroscience	Germany, Canada, Switzerland	Review of 60 studies	Variable	Variable	Variable
Chivu A	EEG Microstates in Mood and Anxiety Disorders: A Meta-analysis	Romania, Czech Republic	Meta-Analysis of 12 studies 787 subjects	Variable	Variable	Variable
Zanesco A	Normative Temporal Dynamics of Resting EEG Microstates	USA	Meta-Analysis of 93 studies 6583 subjects	Variable	Variable	Variable
Koenig T	EEG-Meta-Microstates: Towards a more objective use of resting-state EEG microstate findings across studies	Switzerland, Sweden, USA, UK, Italy, Czech Republic, Germany, China	Meta-Analysis of 40 studies	Variable	Variable	MSTemplateExplorer
Kalburgi SN	MICROSTATELAB: The EEGLAB toolbox for resting-state microstate analysis	Switzerland, USA, Germany, Canada	Tutorial with an example of 34 recordings	64	Variable	MICROSTATELAB
Bagdasarov A	Microstate Analysis of infant EEG: Tutorial and reliability	USA, Switzerland	Tutorial with an example of 55 recordings	128	5	Cartool
Murphy M	A potential source of bias in group-level EEG microstate analysis	USA, China	30	64	5	Cartool
Kleinert T	Normative intercorrelations between EEG microstate characteristics	Germany, Switzerland, Canada	583 542 test-retest	64	5	MICROSTATELAB
Mikutta CA	Electrocorticographic activation patterns during electroencephalographic microstates	Switzerland, USA, Germany, UK	2	19 & intracranial	7	MICROSTATELAB
Kleinert T	On the reliability of the EEG microstate approach	Germany, Switzerland, Canada	583 542 test-retest	64 & 32	5	MICROSTATELAB
Von Wegner F	Complexity measures for EEG microstate sequences - concepts and algorithms	Australia, Germany, Argentina	19	32	4	own
Herrmann G	Propofol reversibly attenuates short-range microstate ordering and 20 Hz microstate oscillations	Germany, Argentina, Australia	20	128	5	own
Wiemers MC	Frequency analysis of EEG microstate sequences in wakefulness and NREM sleep	Germany, Australia, UK	32	32	4 & 5	own
Diezig S	EEG Microstate Dynamics Associated with Dream-like Experiences during the Transition to Sleep	Switzerland	40	64	5	MICROSTATELAB
Ding X	Electroencephalography microstate class D is a brain marker of subjective sleep quality for college students with high habitual sleep efficiency	China, USA	61	62	4	n.i.
Toplutas E	EEG Microstate Analysis in Patients with Disorders of Consciousness and Its Clinical Significance	Turkey	30	19	4	MICROSTATELAB
Perrottelli A	Electrophysiological correlates of reward anticipation in subjects with schizophrenia: an ERP microstate study	Italy, Switzerland	35 SCZ, 26 HC	32	4 (ERP)	Ragu
Deiber M-P	Resting-state EEG microstates and power spectrum in Borderline Personality Disorder: a high-density EEG study	Switzerland, Italy	16 BPD, 16 HC	256	5	MICROSTATELAB
Jia H	Resting-state EEG Microstate Features Can Quantitatively Predict Autistic Traits in Typically Developing Individuals	China	88	63	4	Cartool
Berchio C	EEG spatial-temporal dynamics of resting-state activity in young women with anorexia nervosa: preliminary evidence	Italy, UK, USA, Denmark	18 AN, 18 HC	256	5	Cartool
Carbone GA	Altered EEG patterns in individuals with disorganized attachment: an EEG microstates study	Italy, Switzerland	50	31	6	Cartool
Takarae Y	EEG microstates as markers for cognitive impairments in Fragile X syndrome	USA	70 FXS, 71 HC	128	6	Cartool
Cao O	Resting-State EEG Reveals Abnormal Microstate Characteristics of Depression with Insomnia	China	63 SD, 32 HC	63	4	MICROSTATELAB
Rubega M	EEG microstates as a signature of hemispheric lateralization in stroke	Italy	51 stroke	30	7	Matlab
Hermans T	Microstate analysis reflects maturation of the preterm brain	Italy, Belgium	48	9	4	MICROSTATELAB
Retsa C	Longstanding Auditory Sensory and Semantic Differences in Preterm Born Children	Switzerland, USA	32	64	8 (ERP)	Cartool
Maurer U	Repetition suppression for familiar visual words through acceleration of early processing	China, Switzerland	18	128	2 (ERP)	Ragu
Bagdasarov A	Exploring the association between EEG microstates during resting-state and error-related activity in young children	USA, Switzerland	90	128	6	Cartool
Penalver-Andres JA	Resting-state functional networks correlate with motor performance in a complex visuomotor task: An EEG microstate pilot study on healthy individuals	Switzerland, Netherlands	36	256	7	MICROSTATELAB
Tomescu M	Personality moderates intra-individual variability in EEG microstate and spontaneous thoughts	Romania, USA	43	128	5	Cartool
Kleinert T	Trait aggression is reflected by a lower temporal stability of EEG resting networks	Canada, Germany	110	64	4	MICROSTATELAB
Artoni	How does Independent Component Analysis preprocessing affect EEG microstates?	Switzerland	216	62	5	Cartool

Legend: n.i. = not indicated; HC = Healthy Controls; SCZ = Schizophrenia; BPD = Borderline Personality Disorder; AN = Anorexia Nervosa; FXS = Fragile X Syndrome; SD = Subclinical Depression; ERP = Event Related Potentials

## Functional Interpretation of Microstate Maps

The first review, (Tarailis et al. 2023), delves into the functional significance of various EEG microstates. Many EEG microstate studies interpret their findings by referencing similar maps from prior publications where functional interpretations were provided. Notably, this recursive referencing has resulted in a limited pool of original papers that aimed to identify the brain networks underlying microstate maps, utilizing methods such as simultaneous EEG-fMRI (Britz et al. 2010) or EEG source imaging (Custo et al. 2017; Bréchet et al. 2019). In their review, (Tarailis et al. 2023) aggregated microstate maps from 50 different studies, categorizing them into seven distinct microstate maps based on spatial similarities. The authors proceeded to outline the experimental or pathological conditions that led to the modulation of each microstate, thus emphasizing the most influential factors. This review not only aids future researchers in interpreting their results, but also contributes to the standardization of microstate map labeling.

Building on the work of (Tarailis et al. 2023), the paper by (Koenig et al. 2023) introduces a MATLAB-based toolbox that generates microstate maps derived from various studies. The toolbox facilitates the calculation of spatial similarities between one’s maps and the reference ones. Additionally, it allows for the extraction of EEG meta-microstates, i.e., microstate maps derived from all studies in the database, which can then be used as template maps for fitting to individually recorded data. Presently, the database incorporates microstate maps from 40 studies, and the authors anticipate its expansion as researchers contribute their microstate maps.

The methodology paper authored by (Murphy et al. 2023) reinforces the concept of employing global template maps for fitting individual data. Through a systematic analysis, the authors demonstrate that fitting individual subgroup maps to the data, rather than utilizing global maps derived from all groups within a study, results in significantly inflated Type I error rates. This discovery strongly advocates using uniform template microstate maps when fitting different experimental groups, whether obtained through global clustering across all study subjects or by employing externally defined meta-microstates as provided by the toolbox developed by (Koenig et al. 2023). Nevertheless, (Murphy et al. 2023) also underscore the importance of acknowledging potential differences in microstate topographies between groups. They propose a paired analysis of all subgroup maps to account for these topographic variations across different experimental groups.

### The Temporal Dynamics of EEG Microstates

The conventional method for extracting the temporal dynamics of EEG microstates involves fitting template maps to individual EEG data by calculating the spatial correlation between the template maps and the recorded map at each time point. Subsequently, a winner-take-all approach is employed to assign a specific template map to each time point, revealing microstates by identifying continuous time periods labeled with a particular template map, typically lasting 30–120 milliseconds (depending on smoothing parameters). This fitting procedure enables the extraction of diverse matrices representing microstates’ presence and temporal dynamics. Traditionally, these matrices depict the average duration of each microstate, the occurrence rate, and the percentage of time coverage.

In a meta-analysis conducted by (Zanesco 2023), the average of these parameters across studies was examined, characterizing the heterogeneity attributed to sampling variability, developmental differences, clinical diagnoses, or methodological factors. The analysis encompassed an impressive number of 93 studies, involving a total of 6583 subjects from diverse populations. The findings affirm an average microstate duration of approximately 30–120 milliseconds, with substantial heterogeneity observed across study samples and the prevalence of microstate C in all temporal parameters. This study furnishes robust estimates of average microstate dynamics, offering crucial guidance to researchers analyzing their data by providing estimates of the plausible range of values for microstate temporal parameters.

An intriguing aspect of the temporal characteristics of EEG microstates lies in their intercorrelation, specifically the interactions among the networks underlying these microstates. (Kleinert et al. 2023a) delved into this question, investigating intercorrelations in a substantial dataset comprising 583 subjects. Additionally, they explored the test-retest reliability of these intercorrelations by conducting a retest session with the same subjects after an average interval of 63 days. Notably, the five microstate maps identified in this study corresponded to the meta-microstate maps reported by (Koenig et al. 2023), affirming the consistency of results across datasets. In parallel with Zanesco’s meta-analysis (Zanesco 2023), this investigation highlighted the predominance of microstate C. However, it also unveiled significant dependencies in the temporal characteristics among different microstates, particularly a mutually reinforcing relationship between microstates A and B. Notably, these microstates are predominantly associated with sensory processing, as outlined in the work by (Tarailis et al. 2023).

In a subsequent study utilizing the same two datasets, (Kleinert et al. 2023b) shifted their focus to the test-retest reliability of the temporal parameters of microstates. Subjects were not only measured twice with a 63-day interval (*N* = 583 in the first and *N* = 542 in the second session) but also underwent measurements twice within the same day, enabling the authors to assess both short- and long-term test-retest reliability. The analysis revealed excellent topographic map consistency when extracting five microstate maps instead of only four maps. This is a crucial finding affirming previous studies cautioning against extracting four maps based solely on tradition without considering objective criteria (Custo et al. 2017). The primary discovery of the study was the remarkable short-term test-retest reliability of microstate durations, occurrences, and coverages, along with good long-term retest reliability coefficients over an average interval of 63 days. Notably, there was no discernible systematic decrease in retest reliability with an increasing interval, providing compelling evidence supporting the idea that microstate dynamics represent stable neural traits, as suggested by prior research (e.g., (da Cruz et al. 2020; Murphy et al. 2020; Zanesco et al. 2020).

It is widely accepted that EEG microstates represent global patterns of relatively slow and highly synchronized oscillations that modulate interactions between local brain structures, predominantly active in the higher frequency (high gamma) range (Koenig et al. 2005; Michel and Koenig 2018). Despite this consensus, the direct link between EEG microstates and local changes in high-gamma cortical oscillations has not been experimentally demonstrated. Addressing this question, (Mikutta et al. 2023) conducted a study combining scalp EEG microstate analysis with electrocorticography (ECoG) and stereotactic EEG (SEEG), designed to capture local brain activity. In two patients undergoing preoperative epilepsy evaluation with intracranial EEG, simultaneous scalp EEG was recorded and analyzed using the microstate approach. The temporal-spatial evolution of intracranial signals was analyzed across different frequency bands, and the covariance with the time course of a given microstate class was calculated. This analysis revealed a significant covariation of ECoG/SEEG spectral amplitudes with microstate timelines across all four frequency bands in both patients. This result presents the first experimental evidence demonstrating a correlation between global scalp EEG microstates and ECoG/SEEG field potentials.

### The Complexity of Microstate Sequences

The study conducted by (Kleinert et al. 2023a) extended its examination to the reliability of an additional measure of the temporal dynamics of microstates—the transitions between microstates—utilizing first-order Markov models. The findings revealed modest test-retest reliability for these measures, confirming von Wegner’s observation (von Wegner et al. 2017) that microstate sequences exhibit non-Markovian processes. Moreover, previous work by (van de Ville et al. 2010) demonstrated long-range dependencies that are incompatible with first-order Markov models. A recent study by (Artoni et al. 2023) presented more intricate microstate sequences that cannot be adequately captured by first-order transitions alone. Assessing the complexity of microstate sequences offers an intriguing, yet unexplored, avenue to investigate whether there are underlying rules or some form of syntax within microstate sequences or whether they inherently follow a random pattern. (von Wegner et al. 2023) compared various complexity measures and their application to EEG microstates. They evaluated entropy-based methods, fractal dimension, and Hurst exponent analysis using wake and sleep EEG data. The outcomes of this systematic evaluation of diverse measures contribute significantly to future discussions about the complexity inherent in microstate sequences.

In the study conducted by (Wiemers et al. 2023), time-series analyses were employed to investigate microstate sequences during wakefulness and various stages of non-REM sleep, aiming to understand their effects on distinct time scales. Markov tests were utilized for very short time scales, entropy rate analysis was applied to intermediate time scales, and spectral analysis of microstate sequences was conducted for long time scales. The entropy analysis revealed that microstate sequences exhibit increased predictability (i.e., reduced randomness) with the transition from wakefulness to sleep. In contrast, periodic microstate behavior was identified across all states, albeit associated with different EEG frequencies. Specifically, alpha frequencies were observed during wakefulness, theta frequencies in N1, sleep spindle frequencies in N2, and delta frequencies in N3. These findings contribute to our understanding of the dynamic changes in microstate sequences across different states of consciousness and their association with specific EEG frequencies.

An important message regarding the analysis of microstate sequences is provided in the paper of (Hermann et al. 2024). This study examined the effect of propofol on microstate sequences and uncovered that resting-state sequences exhibited a periodicity of 20 Hz (twice the dominant alpha frequency). This periodicity increased after propofol administration, accompanied by a higher entropy rate. This observation aligns with an earlier study by (Artoni et al. 2021), which demonstrated a U-shaped microstate complexity behavior with increasing propofol doses. The important finding of the paper of (Hermann et al. 2024) is that these effects were masked when the microstate maps were fitted solely to the global field power peaks, an approach still commonly employed in microstate studies.

### Microstates and Consciousness

The studies conducted by (Wiemers et al. 2023) on sleep and (Hermann et al. 2024) on the effects of propofol have provided valuable insights into the influence of the level of consciousness on the complexity of EEG microstate sequences. These findings corroborate earlier reports by (Artoni et al. 2021, 2023) that suggested a connection between microstate complexity and consciousness levels. Moreover, research has demonstrated that conventional descriptors of the temporal dynamics of microstates are modulated by sleep (Brodbeck et al. 2012; Brechet et al. 2020), anesthesia (Artoni et al. 2021; Li et al. 2021; Lapointe et al. 2023), and various states of consciousness (for a comprehensive review, see (Brechet and Michel 2022). This special issue contributes new data to the understanding of the sensitivity of EEG microstates to the level of consciousness. (Diezig et al. 2022) specifically examined changes in EEG microstates during the transition to sleep, a period marked by uncontrolled thinking and perceptual imagery, commonly known as hypnagogic imagery. Their findings revealed that these dream-like experiences, occurring while still awake, were associated with an increased presence of a microstate originating from the superior and middle frontal gyrus and precuneus. Simultaneously, a decrease in activity was observed in higher-order visual areas—a result consistent with observations in subjects reporting dreams during NREM sleep (Brechet et al. 2020). This research adds valuable nuances to our understanding of the relationship between EEG microstates and the varying states of consciousness.

While specific microstate classes have been associated with sleep or the transition to sleep, the study conducted by (Ding et al. 2023) explored the intriguing possibility of using microstates in awake resting EEG as an indicator of sleep quality. Establishing an objective marker for sleep quality could be particularly valuable in clinical studies involving patients with intellectual disabilities who may have difficulty reliably reporting their sleep quality. In their investigation involving 63 college students, (Ding et al. 2023) discovered a positive correlation between sleep quality reports and the occurrence of class D microstates, especially in the high habitual sleep efficiency group. Notably, this microstate has been previously shown to increase during sleep (Brechet et al. 2020).

Adding to this perspective, (Toplutas et al. 2023) observed substantial differences in the coverage of microstate class D between vegetative and minimally conscious patients in their study on microstates in 28 patients with disorders of consciousness. Moreover, they identified strong correlations between microstate D`s characteristics and clinical scale scores. The findings from this study, and the sleep-related investigations mentioned earlier, suggest that the Class D microstate could potentially serve as a candidate biomarker for assessing the level of consciousness. This opens avenues for further research into the clinical utility of microstate analysis in understanding and monitoring states of consciousness.

### Microstates in Neurological and Psychiatric Disorders

The sensitivity of EEG microstate characteristics of neuropsychiatric diseases has been demonstrated in numerous studies, particularly in patients with schizophrenia (Koenig et al. 1999; Khanna et al. 2015; Rieger et al. 2016; da Cruz et al. 2020) and mood disorders (Damborska et al. 2019; Murphy et al. 2020). In this issue, (Chivu et al. 2023) present a meta-analysis of microstate characteristics in mood and anxiety disorders, including 12 studies with a total of 787 subjects. Effect sizes were estimated using Hedges’ g, with age, gender, medication, and comorbidities considered as moderators. The analysis revealed a significant increase in the occurrence of microstate B when considering both pathologies, with a small effect size (Hedges’ g = 0.35). Specifically, when examining only studies comparing mood disorders to healthy controls, the effect size of microstate B increased (g = 0.41), and a significant decrease in microstate D occurrence was noted (g = -0.34). In the context of the potential functional interpretation of microstate B, summarized in the review paper by (Tarailis et al. 2023), as described earlier, this result may suggest that patients with mood and anxiety disorders focus their thoughts more on visual processing related to the self, self-visualization, and autobiographical memory compared to healthy controls. This finding adds to the growing body of evidence supporting the use of EEG microstate analysis as a sensitive tool for understanding and characterizing neuropsychiatric disorders.

However, conflicting findings were reported by (Cao et al. 2023), who investigated EEG microstates in a sample of sub-clinically depressed patients (32 individuals experiencing insomnia and 31 without) and 32 healthy controls. Intriguingly, the study found a significantly reduced occurrence of microstate B in both patient groups compared to controls, which was more pronounced in patients with insomnia. This discovery stands in contrast to the meta-analysis discussed earlier and implies that additional research directly comparing different subtypes of mood disorders at various severity levels is essential for a comprehensive understanding of EEG microstate patterns in these conditions.

The study conducted by (Deiber et al. 2023) delved into microstate and EEG frequency alterations in 16 patients with borderline personality disorder (BPD) in comparison to 16 age-matched controls. The findings revealed a contrasting prevalence of microstate class C (lower than controls) and class E (higher than controls) in individuals with BPD. Additionally, the occurrence of microstate C showed a positive correlation with global alpha performance and a negative correlation with global delta performance. From these observations, the authors inferred that individuals with BPD demonstrate a state of cortical hyperactivation, aligning with symptoms indicative of heightened arousal and/or vigilance.

Microstates in Autism Spectrum Disorder (ASD) have been the focus of extensive research, with a recent review by (Das et al. 2022) highlighting a consistent finding of decreased microstate C in individuals with ASD. In this special issue, (Jia et al. 2023) assessed whether EEG microstate features can serve as predictors for Autism Spectrum Quotient (AQ) questionnaire scores in a sample of 88 healthy subjects. Employing the Least Absolute Shrinkage and Selection Operator (LASSO) algorithm along with correlation analysis, the researchers identified four crucial microstate features that could be utilized to predict autistic traits. Using machine learning methods, they demonstrated that the predicted autistic trait scores, based on these four microstate features, exhibited significant agreement with self-reported scores from the AQ questionnaire. This research contributes valuable insights into the potential use of EEG microstate features as predictors for autistic traits in the general population.

In a similar fashion, based on questionnaires in healthy individuals, (Carbone et al. 2024) examined whether microstate characteristics can differentiate individuals with high and low Disorganized Attachment (DA) scores. They scored 50 college students before and after a highly stressful 1 ½ hours Adult Attachment Interview, which allowed them to group the subjects into “organized/resolved” and “disorganized/unresolved” groups. The results showed differences in duration and occurrence in specific microstates in the two groups. Interestingly, significant microstate changes were also found pre- and post-interview for both groups, indicating changes in microstate parameters due to the pre-rest state condition.

(Takarae et al. 2023) examined EEG microstates in 70 patients with Fragile X syndrome, a genetic disorder that causes mild to severe intellectual disabilities, and compared them with 70 healthy controls. They reported an increased presence of microstates C and D in the patient group, which was more pronounced in males. These findings led the authors to conclude that these changes in specific microstates are likely related to two of the most prominent cognitive features of Fragile X syndrome: intellectual disability and attention impairments and suggest that microstate parameters could serve as markers to study cognitive impairment and evaluate treatment outcomes in this population.

(Berchio et al. 2023) present the first study investigating EEG microstate changes in young women with anorexia nervosa. The study compared 18 patients with 18 healthy controls. The main difference observed between the groups was a reduced time coverage of microstate C and an increased time coverage and occurrence of microstate E in patients compared to controls. As a methodological aspect, the study also directly compared the results of fitting subgroup microstate maps or overall group mean maps, confirming the earlier finding by (Murphy et al. 2023) that these two approaches lead to different significant results, and fitting global mean maps is preferable.

(Hermans et al. 2023) present a challenging study in which they repeatedly recorded the EEG of 48 premature infants at different postmenstrual ages. Despite the EEG being recorded from only 9 channels, microstate analysis was applied. Across all subjects, four microstate maps were identified, demonstrating non-random syntax and accounting for about 70% of the global variance. Notably, these four maps exhibited consistent topography throughout infant aging, bearing striking similarities to canonical adult microstate maps despite the limited number of recording electrodes. The study revealed that all microstates decreased in duration and increased in frequency with age. Additionally, the Hurst exponent of each microstate sequence decreased with age. These unique findings may directly reflect the development and formation of neural networks in the preterm brain, suggesting that microstate EEG analysis holds promise as a tool for monitoring the maturation of the preterm neonatal brain.

In the study by (Retsa et al. 2023), the long-term consequences of pre-term birth on auditory processing were investigated. The researchers recorded auditory event-related potentials (ERP) to environmental sounds (living vs. manufactured objects) in 10-year-old children born at term or very preterm. ERP microstate analysis was applied to the ERPs. Microstate analysis of the ERPs exhibited similar basic characteristics to resting-state EEG analysis: potential map configurations remained stable for a period of time, typically covering the duration of traditional ERP components (Brandeis and Lehmann 1986; Michel et al. 2001). The main difference in the analysis method is that polarity information is considered relevant in ERP analysis, whereas it is ignored in the spontaneous EEG (Murray et al. 2008). Additionally, parameters like the onset and offset of each microstate after stimulus onset become relevant temporal parameters (Michel et al. 1999). Using this approach, (Retsa et al. 2023) revealed significant differences between the two groups during early auditory sensory processing as well as during later semantic processing of the sounds. Moreover, the distinct brain activity patterns between the two semantic categories could reliably classify children according to their preterm status.

In another study by (Rubega et al. 2023), EEG microstates were examined in 51 first-ever ischemic stroke survivors, 24 with right-hemisphere strokes and 27 with left-hemisphere strokes, who underwent resting-state EEG recording in the acute and subacute phases. The study found that EEG microstate topographies were quite similar between right- and left-hemisphere lesioned patients and resembled those of healthy subjects, except for one microstate map that differed between the two groups of patients. Regarding the temporal dynamics of the canonical microstates, microstate D showed differences between right- and left-hemisphere stroke patients.

In the study by (Perrottelli et al. 2023), ERP microstate analysis was used to investigate reward anticipation in individuals with schizophrenia. They examined ERP microstate changes during a monetary incentive delay task, where reward, loss, and neutral cues were presented. The study included 30 individuals with schizophrenia and 23 healthy controls. The researchers observed changes in the first (125–187 ms) and second (261–414 ms) anticipatory cue-related microstate classes in patients compared to healthy controls. The conclusion was that abnormalities in ERP microstates can be detected in the early stages of reward processing, suggesting that these dysfunctions may reflect an impaired effective evaluation of incoming pleasant experiences.

### State and Trait Relations of Microstates

The review by (Schiller et al. 2023) synthesizes findings from 60 studies that employed the EEG and ERP microstate approach to investigate socio-affective states (e.g. (Schiller et al. 2016) or traits (e.g. (Schiller et al. 2019) in the brain. Social interaction necessitates the rapid processing of diverse socio-affective signals and their integration with social knowledge and expectations at the millisecond time scale accessible to EEG/ERP microstate analysis. The review highlights how the microstate approach provides a unique capability for identifying, timing, sequencing, and quantifying the activation of large-scale brain networks relevant to our socio-affective mind.

In the study conducted by (Kleinert and Nash 2022), the investigation focused on whether individual differences in trait aggression are reflected in resting-state EEG microstates. The researchers recorded the EEG of 101 participants at rest and examined the correlation between the temporal dynamics of the microstates and scores from an aggression questionnaire featuring various aggression subscales. Notably, the study utilized the recently introduced term “microstate stability,” with higher stability represented by longer lasting and less frequently occurring microstates and recently associated with higher levels of trait self-control (Kleinert et al. 2022). The findings indicated a negative association between the stability of all four microstates and aggression, with contributions from the physical aggression, verbal aggression, and hostility subscales. In contrast, no such association was observed with the anger subscale. This negative relationship between microstate stability and aggression was predominantly observed in males and, to a lesser extent, in females.

In the study conducted by (Tomescu et al. 2023), the investigation focused on the fluctuation of spontaneous thoughts and EEG microstate dynamics across two days, examining their relationship with personality traits. The researchers recorded the resting-state EEG of 43 participants on two separate days. Participants completed the Amsterdam Resting-State Questionnaire, a self-report tool quantifying mind-wandering across ten scales, and the Neo Personality Inventory, assessing the “Big Five” personality traits (extraversion, agreeableness, openness, conscientiousness, and neuroticism). The results revealed significant daily changes in the associations between EEG microstates and spontaneous cognition. Furthermore, personality traits were linked to these day-to-day variations in both microstates and spontaneous thoughts.

The study conducted by (Bagdasarov et al. 2023) aimed to explore the potential link between individual differences in resting-state intrinsic brain activity and external information processing. The researchers investigated the relationship between resting-state EEG microstates and the ERP microstate of error-related negativity during a go/no-go task in a cohort of 90 children aged 4–8 years. Preschool anxiety, withdrawal, and approach tendencies were assessed through parent-report questionnaires. In the ERP analysis, the researchers observed the anticipated topography of the event-related negativity (ERN) component, finding higher global field power (GFP) in children with an increased risk for anxiety. Interestingly, they discovered a correlation between the GFP of the ERN microstate and a specific resting EEG microstate characterized by a frontal-central scalp topography. Additionally, the source localization results indicated an overlap between the neural generators of the error-related microstate during the task and this resting-state microstate. These shared brain regions were associated with higher-order cognitive processes involved in error processing.

The study by (Penalver-Andres et al. 2022) investigated the relationship between person-specific pre-task resting-state activity and subsequent performance in a motor task. The researchers recorded the resting EEG of 36 healthy volunteers before they engaged in a virtual surfing task. Analyzing microstate features and correlating them with motor behavioral metrics, they observed that the pre-activation of microstate C, associated with the posterior default mode network (DMN), correlated with poorer performance in the subsequent motor task. However, the hypothesized positive correlation between microstate D, linked to the dorsal attention network, did not reach significance.

Finally, (Maurer et al. 2023) conducted an ERP microstate study to explore the repetition effects of familiar visual words in a Chinese population and elucidate the relationship between the visual N1 component and a later appearing N200 component specific to word repetition in Chinese. Utilizing the microstate approach, which enables the assessment of the onset and offset of ERP components (parameters oftentimes overlooked in conventional ERP peak analysis), they revealed that the previously reported N200 repetition effect is, in fact, a result of the shortening of the N1 offset. This insight, uniquely provided by the ERP microstate analysis (Michel et al. 1999), contributes to a more refined understanding of the neural dynamics involved in word repetition effects.

### Microstate Analysis Tools

Several freely available analysis tools are available to extract EEG microstates. Some of them are stand-alone programs (LORETA-KEY by Pascual-Marqui ((Pascual-Marqui et al. 1995); http://www.uzh.ch/keyinst/loreta; CARTOOL by Brunet and Michel (Brunet et al. 2011); https://sites.google.com/site/cartoolcommunity/; others are tools based on the MATLAB programming platform (Microstate EEGLAB toolbox by Poulsen (Poulsen et al. 2018) https://github.com/atpoulsen/Microstate-EEGlab-toolbox), the EEGLAB plugin MICROSTATELAB by Koenig (Nagabhushan Kalburgi et al. 2023), and the + Microstate by Tait & Zhang ((Tait and Zhang 2022); https://github.com/lukewtait/microstate_toolbox. Furthermore, a Phyton-based toolbox integrated in MNE has recently been published (Pycrostates by Ferat (Férat et al. 2022); https://pycrostates.readthedocs.io/en/latest/. According to the review by (Zanesco 2023) published in this issue, the most commonly reported software used by authors to perform topographic clustering and microstate quantification were the MICROSTATELAB toolbox and CARTOOL (see also Table 1). In this issue, tutorials for these two programs are provided. (Nagabhushan Kalburgi et al. 2023) introduce the MICROSTATELAB toolbox in a step-by-step tutorial, using a sample dataset of 34 publicly available resting-state EEG recordings to illustrate the different steps provided by the toolbox. The CARTOOL tutorial by (Bagdasarov et al. 2024) focuses on microstate source localization, including MRI preprocessing, brain and gray matter extraction, and electrode co-registration. They illustrate the different steps with data from young infants made publicly available in the BIDS format. In addition, the authors have added a step-by-step tutorial and an accompanying website for performing microstate analysis as supplementary material: https://github.com/gaffreylab/EEG-Microstate-Analysis-Tutorial. Hopefully, these tutorials will improve the methodological standards of microstate research and provide the community with full functionality of microstate analysis.

The existing literature needs a standardized pipeline for the preprocessing of raw EEG data for microstate analysis. Variations exist in bandpass filters, sampling rates, bad electrode interpolation methods, and artifact detection and removal techniques across studies. An essential consideration is the application of Independent Component Analysis (ICA) for artifact detection and removal, where some studies use ICA solely for eye movement removal, while others remove multiple components perceived as non-brain activity. (Artoni and Michel 2024) systematically investigated the impact of ICA-based artifact removal on microstate analysis using a normative resting-state EEG dataset with eyes open and eyes closed recordings. Four artifact removal strategies with ICA were tested: no removal, only ocular artifact removal, removing all reliably identified physiological/non-physiological artifacts, and retaining only reliably identified brain ICs. The findings demonstrated that excluding more than just eye movement IC components had no effect on microstate topographies and temporal features, highlighting the robustness of microstate topographies and features to a variety of artifacts.

## Conclusions

EEG microstate analysis has matured into a robust methodology for assessing large-scale, whole-brain network dynamics. The reviews and meta-analyses in this special issue not only highlight the substantial number of publications on this methodology, but also reveal a considerable consensus on the functional interpretation of EEG microstates. However, they also indicate that several inconsistencies persist, partly due to methodological variations and underpowered studies. Larger multicenter studies with clearly defined experimental designs, subject selection, and analysis methods are needed, especially for clinical studies. Concerning analysis pipelines, step-by-step tutorials, as presented in this special issue, prove to be valuable. Additionally, it would be crucial to conduct a study comparing different available analysis tools and assessing the impact of core analysis parameters, such as the number of microstates to fit or fitting thresholds, on a large publicly available dataset.

In conclusion, the articles in this 3-part special issue, coupled with previously published studies, illustrate that EEG microstate analysis is a powerful tool, which uniquely provides information on the temporal dynamics of brain networks with high sensitivity and specificity. This makes it a promising method for identifying candidate biomarkers for various pathologies and eventually predicting and evaluating treatment success. In this context, we are also delighted to announce that we plan to hold a follow-up conference dedicated to microstate analysis on July 9–12, 2024, in Geneva (see www.microstate-conference.com for details).

## Data Availability

No datasets were generated or analysed during the current study.
